# The ins and outs of telomere crisis in cancer

**DOI:** 10.1186/s13073-018-0596-4

**Published:** 2018-11-27

**Authors:** Patrick von Morgen, John Maciejowski

**Affiliations:** Molecular Biology Program, Sloan Kettering Institute, Memorial Sloan Kettering Cancer Center, New York, NY 10065 USA

## Abstract

Telomere crisis is linked with many of the genomic alterations found in cancer genomes. A new understanding of how these alterations arise points towards an active role for innate immune sensors during crisis and to new opportunities for the treatment and diagnosis of cancer.

## Telomere crisis shapes the cancer genome

Telomeres protect genomic integrity by masking natural chromosome ends from the DNA damage response and repair pathways. The erosion of telomeric sequence due to incomplete replication compromises this protection with broad consequences for aging. In the context of cancer, telomere shortening can exert a tumor-suppressive effect by enforcing a proliferation arrest. On the other hand, telomere deprotection can also enable cancer growth through telomere crisis, a state of extensive genomic instability and cell death. Telomere crisis causes numerous, cancer-relevant genome alterations, including translocations, amplifications, and deletions, and has now been linked with the genesis of the mutational phenomena chromothripsis and kataegis [1]. Chromothripsis is characterized by clusters of chromosome rearrangements that occur in a single event. Although initially estimated to be present in only ~ 3% of cancers, revised estimates suggest that chromothripsis is pervasive, with a frequency > 50% in several cancer types [2]. Kataegis is defined by the presence of clusters of cytosine mutations, which are hypothesized to be caused by APOBEC3 (apolipoprotein B mRNA-editing enzyme, catalytic polypeptide-like 3)-catalyzed cytosine deamination [3].

## Sources of DNA damage in telomere crisis

**Fig. 1:**
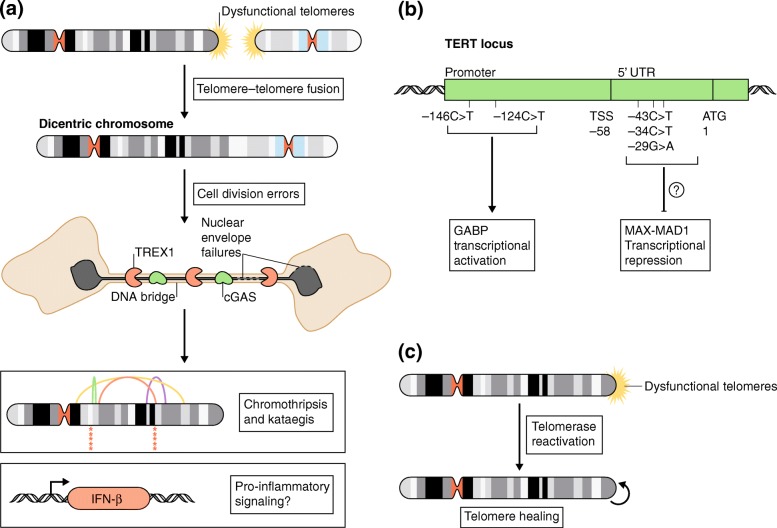
**a** Dicentric chromosomes are formed by the fusion of dysfunctional telomeres during telomere crisis. Intact dicentrics persist through mitosis and form DNA bridges, which induce nuclear envelope failures resulting in the accumulation of Three prime repair exonuclease 1 ( *TREX1* ) and cyclic GMP-AMP synthase ( *cGAS* ) on the bridge DNA. Parts of the dicentric chromosome that are present in the DNA bridge undergo TREX1-mediated resection and extensive fragmentation. The fragmented chromosome undergoes haphazard repair, yielding a highly rearranged chromothripsis chromosome. Chromothripsis-associated breakpoints frequently display kataegis. Nuclear envelope failures at DNA bridges may result in a cGAS-dependent pro-inflammatory response. **b** Mutations in the telomerase reverse transcriptase ( *TERT* ) promoter drive telomerase reactivation through the creation of a GABP transcription factor binding site. Recently identified, recurrent mutations in the TERT 5′ untranslated region ( *UTR* ) associate with longer telomere length in clear cell renal cell carcinomas and are hypothesized to induce telomerase reactivation via inactivation of MAX-MAD1 transcriptional repression. **c** Telomerase reactivation heals dysfunctional telomeres to restore genomic stability and to provide a path out of telomere crisis

During telomere crisis, the aberrant activation of DNA repair pathways at natural chromosome ends results in telomere–telomere fusion and the creation of dicentric chromosomes (Fig. 1a). Although dicentric chromosomes are recognized as precipitants of instability, the precise mechanisms that give rise to genomic alteration during telomere crisis are not well understood. Intact dicentric chromosomes persist throughout mitosis and develop into DNA bridges [1]. DNA bridges trigger nuclear envelope rupturing in cells that have dicentric chromosomes, leading to their partial degradation by Three prime repair exonuclease 1 (TREX1), a cytosolic exonuclease that clears cytosolic DNA to prevent autoimmunity.

Nuclear envelope rupturing may be an important driver of genomic alteration during telomere crisis. Outside of telomere crisis, nuclear envelope rupturing occurs in micronuclei, aberrant nuclear structures formed after errors in cell division, where it has been linked with DNA damage and chromothripsis [4, 5]. Primary nuclei can also undergo nuclear envelope rupturing in the context of p53 or Rb deficiency or during cell migration through tight constrictions [6]. Loss of nuclear compartmentalization in this context induces immediate DNA damage. The causes of nuclear envelope failure during telomere crisis are not known but may derive from insufficient lamina coating, nuclear compression or, as recently suggested, insufficient integration of specific nuclear envelope proteins [4].

The induction of APOBEC3 mutagenesis is another threat to genomic integrity during telomere crisis. The APOBEC3s are a family of cytosine deaminases that specifically target single-stranded DNA as part of their normal function as anti-viral restriction factors. An APOBEC3-linked mutational signature is found in at least 22 cancer types, in which it can occur at high frequency. APOBEC3 mutations often cluster at rearrangement breakpoints, where they are termed kataegis clusters, or they can be dispersed throughout the genome. Observations to date suggest that APOBEC3 mutagenesis during telomere crisis is limited to kataegis clusters. The cause of APOBEC3 dysfunction during crisis is not known but may be related to the observed accumulation of single-stranded DNA at DNA bridges [1]. Likewise, the relationship between APOBEC3 mutagenesis and rearrangement breakpoints is not well-defined but may reflect an active role for APOBEC3-dependent deamination in triggering DNA double-strand breaks and consequent chromosome rearrangements.

It is likely that TREX1 is not the only cytosolic factor to engage genomic DNA during telomere crisis. Loss of function mutations in the *Trex1* gene cause Aicardi-Goutières syndrome, which is characterized by elevated type I interferon levels and severe encephalitis. These symptoms have been linked with activation of the cyclic GMP-AMP synthase (cGAS)-stimulator of interferon genes (STING) cytosolic DNA-sensing pathway, which detects cytosolic DNA and triggers a wide-ranging, anti-viral response that includes the induction of type I interferons and other pro-inflammatory genes. The cGAS-STING pathway senses and responds to cytosolic DNA species that accumulate as a result of genomic instability by activating an IRF3- and NFκB-dependent pro-inflammatory, transcriptional response. This inflammatory response can induce senescence and even apoptosis and thus have pronounced effects on cancer cells. The full impact of this inflammatory response in a clinical setting is not known, but there is evidence that it can influence the efficacy of radio- and immunotherapies. We speculate that the cGAS-STING pathway promotes replicative senescence and limits escape from telomere crisis. In support of this view, cGAS, the DNA-sensing component of this pathway, has been previously observed at DNA bridges [7].

## Telomerase activation: the path out of crisis

Genomic rearrangements that are induced during telomere crisis may contribute to carcinogenesis by driving genetic change, but these alterations cannot accumulate indefinitely because they would eventually inhibit cancer growth. Escape from telomere crisis requires re-activation of the telomerase reverse transcriptase (TERT), which is normally silenced during development. Telomerase can enable escape from telomere crisis by synthesizing telomeric repeats de novo at chromosome ends, thus healing shortened telomeres and restoring the capacity for DNA proliferation.

The identification of activating mutations in the TERT promoter point towards one major mechanism of telomerase reactivation. MSK-IMPACT, a large-scale clinical sequencing initiative, has identified these mutations as the most frequent non-coding mutations in cancer [8]. Despite this prevalence, TERT promoter mutations are not sufficient to prevent telomere attrition and the generation of critically short and unprotected telomeres [9]. Instead, TERT promoter mutations sustain cellular lifespan by healing only the shortest telomeres, but cannot indefinitely prevent telomere fusion and genomic instability. Exit from telomere crisis requires further telomerase upregulation.

A recent analysis of clear cell renal carcinomas has identified three additional frequently appearing mutations, which are independent of the highly recurrent TERT promoter mutations, in the TERT 5′ untranslated region [10] (Fig. 1b). The presence of these mutations significantly correlates with increased telomere length, suggesting that these mutated sites also lead to telomerase reactivation (Fig. 1c). All of the identified mutations are located in or near a predicted binding site for the MYC-MAX-MAD1 family of proteins. Although the precise consequence of these specific mutations is not known, it is tempting to speculate that they lead to telomerase expression through transcriptional activation.

## Conclusions and future directions

Telomere crisis is associated with a nearly comprehensive list of genomic alterations. Progress in the field will require mechanistic work to determine the sources of DNA damage during crisis and how these sources contribute to distinct genomic consequences. The data point towards nuclear envelope rupturing as a significant mediator of instability during crisis. In the future, it will be interesting to determine whether the DNA damage that results from nuclear envelope rupturing at DNA bridges, micronuclei, and the nucleus occurs through distinct or similar mechanisms in each of these locations.

In addition to this hypothesized role in genomic rearrangement, nuclear envelope failure at DNA bridges may prove to be significant during telomere crisis as the result of its engagement of other components of the cGAS-STING signaling pathway in addition to TREX1. cGAS and STING have pivotal roles in cancer immunity and in the anti-tumor effects of immune checkpoint blockade. With this in mind, cGAS-STING activation by DNA bridges may promote cancer immunogenicity. We speculate that this pathway could boost the anti-tumor benefits of therapies targeting telomere maintenance. Future work will determine the extent of cGAS-STING activation during telomere crisis and how this activation impacts cell viability, escape from crisis, immune engagement, and potential therapies.

More studies will be required to identify additional mechanisms driving telomerase reactivation in cancer. Telomerase promoter mutations are not sufficient on their own to indefinitely delay replicative senescence and are proposed to work as part of an uncharacterized two-step mechanism [9]. Likewise, telomerase-activating mutations only explain a subset of the telomere maintenance strategies that are active in cancer. Activation of alternative lengthening of telomeres (ALT) plays a role in some cancers and is associated with the cytosolic localization of telomeric repeats and cGAS-STING activation [11]. Therefore, characterizing the mechanism of telomere maintenance in a specific tumor could be important for predicting the course of disease and the potential benefits of treatments affecting telomere maintenance.

Collectively, these recent advances have set the stage for deep insights into how telomere crisis shapes the cancer genome and engages with cytosolic DNA-sensing pathways to alter the course of disease.

## Abbreviations

APOBEC3: Apolipoprotein B mRNA-editing enzyme, catalytic polypeptide-like 3
cGAS: Cyclic GMP-AMP synthase
STING: Stimulator of interferon genes
TERT: Telomerase reverse transcriptase
TREX1: Three prime repair exonuclease 1
